# Synthesis of Biodegradable and Antimicrobial Nanocomposite Films Reinforced for Coffee and Agri-Food Product Preservation

**DOI:** 10.1021/acsomega.3c04017

**Published:** 2023-11-02

**Authors:** Quang Lich Nguyen, Dai Vuong Le, Anh N. Phan, Van Duy Nguyen

**Affiliations:** † School of Engineering and Technology, Hue University, Hue City 530000, Vietnam; ‡ School of Engineering, Chemical Engineering, Newcastle University, Newcastle upon Tyne NE1 7RU, U.K.; § Institute of Biotechnology and Environment, Nha Trang University, Nha Trang 650000, Khanh Hoa, Vietnam

## Abstract

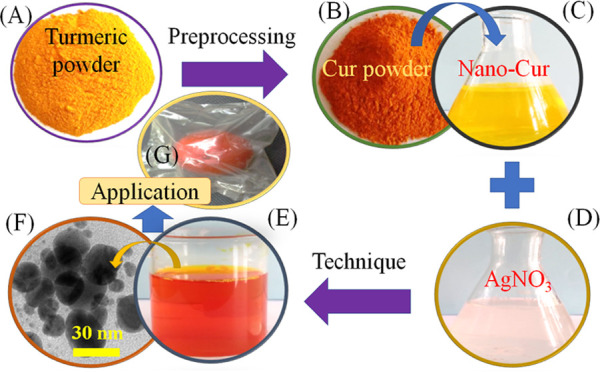

The antimicrobial activity of silver nanoparticles is widely known. However, their application to biodegradable polymeric materials is still limited. In this work, we report a strategy involving the green synthesis of nanocomposite films based on a natural biodegradable matrix. Nanometer-sized silver nanoparticles (*C*-AgNPs) were synthesized with the aid of ultrasound waves between the silver nitrate solution and the nanocurcumin solution. The green synthesized C-AgNPs were found to have particle sizes in the range of 5–25 nm and demonstrated good antimicrobial activity against *Clostridium perfringens*, *Staphylococcus aureus*, *Bacillus subtilis*, *Macrophoma theicola*, and *Aspergillus flavus*. Owing to their physical–chemical and mechanical properties and the excellent antimicrobial activities, the obtained AgNPs were used together with chitosan, cassava starch, and poly(vinyl alcohol) (PVA) to make nanocomposite films, which are suitable for the packaging requirements of various key agricultural and food products such as coffee beans, bamboo straws, and fruits. The nanocomposite films lost up to 85% of their weight after being buried in the soil for 120 days. This indicates that the films made with natural biodegradable materials are environmentally friendly.

## 1 Introduction

Industrial revolution 4.0 has brought about the development of technological platforms associated with safe production and sustainable development in the agriculture and food sectors. The current food industry has had many outstanding improvements and developments, e.g., food nanotechnology.^1^ There has been a shift toward new and innovative applications in the food sector, for example, nanostructured ingredients and food nanosensors.^2^ Food nanotechnology means products made with nanotechnology techniques or tools used in the process of growing, manufacturing, processing, packaging, and protecting food.^3^ This will entail many benefits, such as reduced waste, extended product shelf life, and improved taste. Furthermore, commercial fossil-based polymers are not always environmentally friendly. The serious environmental problems associated with nonbiodegradability of synthetic polymer films has driven research on edible biopolymer films and coatings to replace synthetic polymers for food packaging materials.^4,5^ Recently, there has been increasing interest in the development of polymers of natural origin, namely, biopolymers, due to their low toxicity and biodegradable nature.^6,7^ The combination of biopolymers and nanoparticles, e.g., metal-based or polymeric materials with antimicrobial properties, i.e., alginate, nanocurcumin, and chitosan, has led to an increase in the value and efficiency in the food preservation industry or optoelectronic devices.^8^ Current studies^9,10^ have reported that the integration of silver nanoparticles with other compounds has strong synergistic antimicrobial effects. Polymeric nanoparticles, such as alginate, nanocurcumin, and chitosan, also show antimicrobial activities.^8^ Nanoparticles can be used to make versatile materials for packaging and coating in the food industry.^5,11^ Biodegradable natural polymer films, e.g., starch films, offer alternatives to conventional packaging due to their excellent biodegradability, biocompatibility, renewability, and ease of processing with a wide range of potential applications.^12^ Shende et al.^11^ reported that silver-doped titanium dioxide nanoparticles (NPs) encapsulated with the chitosan–poly(vinyl alcohol) (PVA) film offer very high synergistic antimicrobial activity, which is similar to the study of Usman et al.^13^ for polymer PVA/GO/starch/Ag nanocomposite films. Silver nanoparticles are highly reactive due to a large surface-to-volume ratio. They have a crucial role in inhibiting bacterial growth, which offers higher safety and can prolong the shelf life of foods, conferring great economic benefits.^1,14^

In this work, we fabricated nanocomposite films using silver nanoparticles synthesized from a nanocurcumin solution (adapted from other processes),^9^ chitosan, cassava starch, and PVA. These nanocomposite films were then tested as preservative materials for agricultural and food products (coffee beans, bamboo straws, and fruits) to inhibit bacterial and fungal growth. Biodegradability of these nanocomposite films was also examined.

## 2 Materials and Methods

### 2.1 Green Synthesis of Silver Nanoparticles

**Figure 1:**
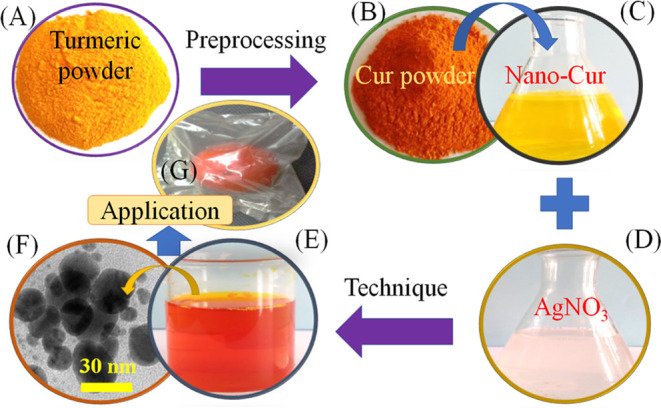
Illustration image of the formation of *C*-AgNPs surrounded by nanocurcumin: (A) turmeric powder, (B) curcumin powder, (C) nanocurcumin, (D) AgNO_3_ solution, (E) *C*-AgNP solution, (F) *C*-AgNP nanoparticles, and (G) biodegradable bags using nanocomposite films.

The procedure for synthesizing silver nanoparticles was adopted from another study^9^ as shown in Figure 1. The fresh turmeric was cut, polished, dried, and ground to obtain turmeric powder (Figure 1A). The dried turmeric powder was mixed with a solvent mixture of 50 mL of acetone and 250 mL of ethyl acetate with the help of ultrasound at a frequency of 28 kHz for 30 min to obtain the crude curcuminoid (Figure 1B). The curcumin nanoparticles were prepared using an oil-in-water emulsion technique (Figure 1C). Next, a reaction of the nanocurcumin solution (15 μg/mL, Figure 1C) and the AgNO_3_ solution (0.02 M, Figure 1D) was carried out to form silver nanoparticles (*C*-AgNPs, Figure 1E) at 30 °C for 30 min. The nanoparticles (*C*-AgNPs, Figure 1F) that have been fabricated can be used for different applications as shown in Figure 1G.

### 2.2 Synthesis of Nanocomposite Films

**Figure 2:**
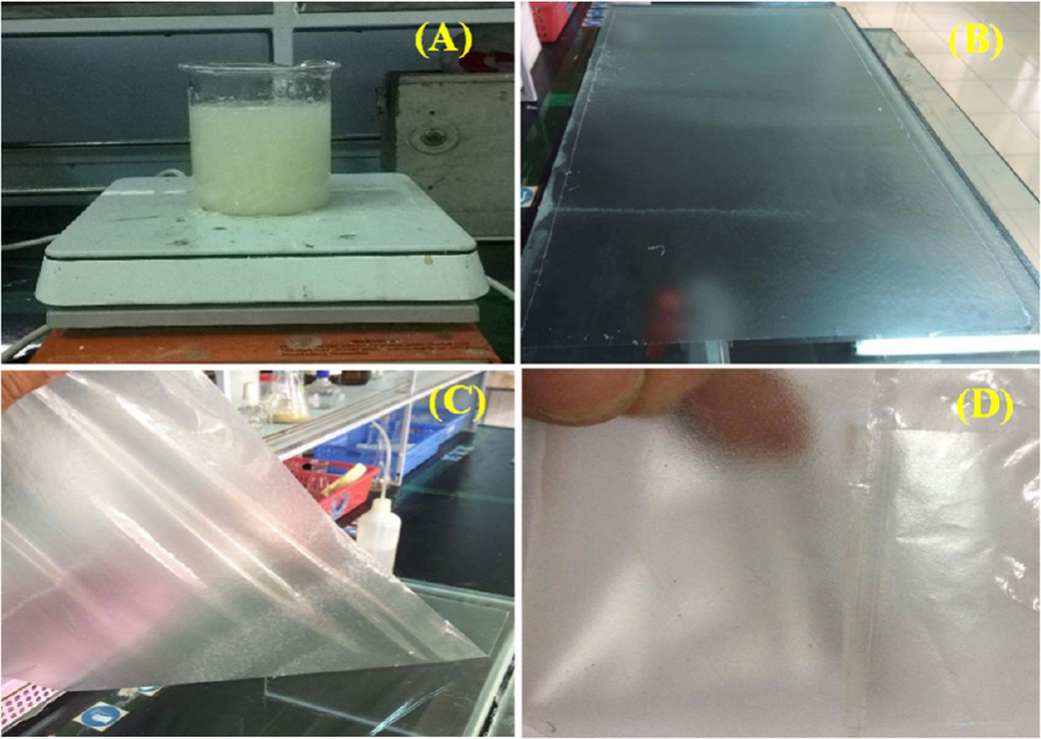
Process of synthesizing nanocomposite films: (A) homogeneous solutions, (B) image of lamination on glass, (C) separating film from glass, and (D) nanocomposite film products.

PVA was mixed in hot water (95 °C) at a ratio of PVA to water of 1:25 (wt/vol). When the PVA was fully dissolved, the temperature of the mixture was reduced to 82 °C (Figure 2A) before adding a known amount of cassava starch (a PVA-to-starch ratio of 2:1 (wt/wt)). Around 1 mL of the chitosan solution (1% (w/v) in 1% acetic acid) was added to the solution. After 20 min of mixing, a known amount of AgNP solution (a ratio of AgNP solution to water of 1:10 vol/vol) with different concentrations (0, 30, and 50 ppm) was added to the solution mixture with continuous stirring for 10 min. The homogeneous solutions (Figure 2A) were cast onto a flat glass plate (Figure 2) and freeze-dried for 24 h at room temperature (28 °C). The obtained products (Figure 2C,D) as the nanocomposite films were designed to apply for the preservation of some agricultural products, which will be discussed in the next section.

### 2.3 Characterization

The optical properties of the *C*-AgNP solution were evaluated using a Shimadzu double-beam spectrophotometer at a resolution of 1 nm between 280 and 600 nm to confirm the formation of nanoparticles. TEM micrographs to determine the size and shape of the synthesized AgNPs were obtained using a JEM 1010-JEOL. The crystal structure of the sintered samples was examined by X-ray diffraction (XRD, D8 Advance) in the 2θ range of 20–70°. Furthermore, Rietveld refinements were performed using FullProf software. The stress–strain curve of the nanocomposite film was determined using the Instron 5967 device (Instron, Norwood, MA).

### 2.4 Antibacterial Assays

The following bacteria strains were used to detect the antimicrobial activity of *C*-AgNPs: *Clostridium perfringens* (ATCC19408), *Staphylococcus aureus* (ATCC6538), and *Bacillus subtilis* (ATCC 23857), which were provided by the Institute of Biotechnology, Hue University. Bacterial strains were spread on a De Man-Rogosa-Sharpe (MRS)-agar (Merck, Germany) plate and incubated at 37 °C for 24 h. Then, a single colony was cultured in 5 mL of MRS at 37 °C for 24 h. 10 μL of culture was spread on an MRS-agar plate, and six wells with 10 mm diameters were created. 50 μL of *C*-AgNPs was dropped into each well, and the plate was incubated at 37 °C for 24 h. The inhibitory activity was assessed as the diameter of the inhibition zone around the well.

To study the antifungal effect of *C*-AgNPs, fungal strains were isolated at the Institute of Biotechnology, Hue University, including *Macrophoma theicola* strains isolated from numerous infected mandarins, *Aspergillus flavus* S3 and *F. oxysporum* A5 isolated from infected corn, and *A. flavus* strains isolated from infected bamboo straws and coffee beans in Thua Thien Hue province. The strains were cultured on potato dextrose agar (PDA) (Merck, Germany) plates with 30 ppm of *C*-AgNPs at 28° for 3 days, and after this period, fungal growth inhibition halos were measured (mm). PDA dishes without *C*-AgNPs were used as controls. To ensure accuracy, each test was conducted three times.

## 3 Results and Discussion

### 3.1 Mechanism of Formation and Characteristics of *C*-AgNPs

**Figure 3:**
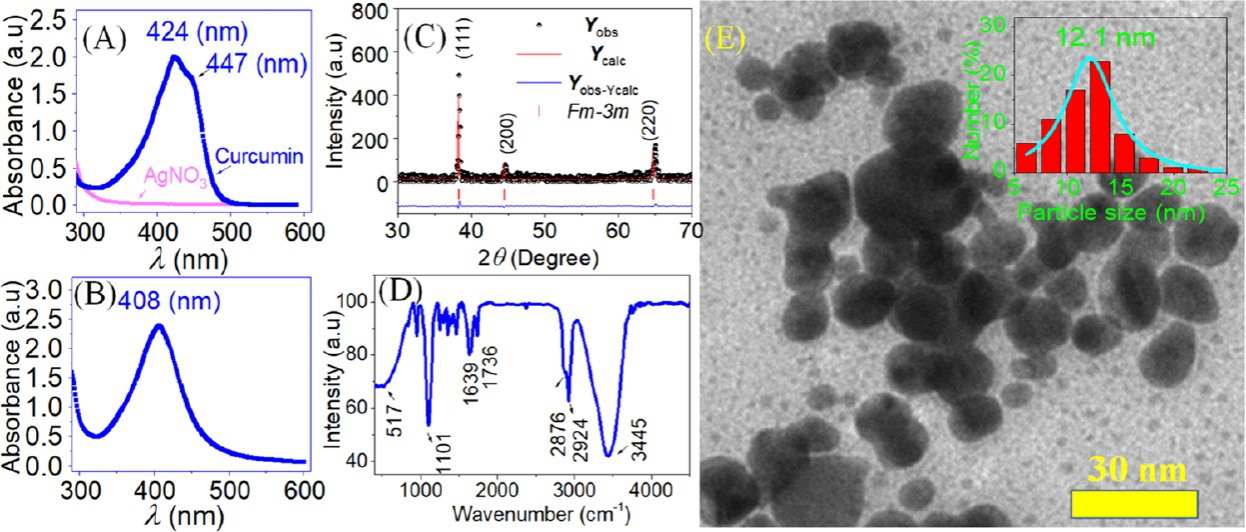
Characteristics of the prepared *C*-AgNPs. (A) UV–vis spectra of the synthesized nanocurcumin and AgNO_3_; (B) UV–vis spectra of the synthesized *C*-AgNPs; (C) XRD pattern of *C*-AgNPs; (D) FTIR band absorption characteristic of AgNPs; and (E) TEM micrographs of *C*-AgNPs.

The formation of *C*-AgNPs using nanocurcumin solution (Figure 1) can explain that the Ag^+^ ions were reduced into Ag° nanoparticles by nanocurcumin through the reaction following the equation^9^
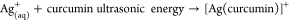
1
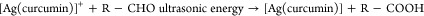
2where Ag_(aq)_^+^ reacted with curcumin solution to form the [Ag(Curcumin)]^+^ complex (eq 1), which reacted with numerous functional groups in the molecular structure of curcumin solution as hydroxyl, carboxyl, amine, and aromatic, to form [Ag(Curcumin)], due to the reduction of Ag^+^ ions through the oxidation of the aldehyde to carboxylic acid groups (eq 2) as reported in another work.^9^ The formation of nanocurcumin was confirmed through ultraviolet–visible (UV–vis) spectral analysis (Figure 3A). The synthesized nanocurcumin features a strong absorption peak at 424 nm and a small shoulder peak at 447 nm.^9^ According to the research results of Subhan et al.,^15^ the UV–vis spectra of the curcumin solution have a maximum absorption band at wavelength 424 nm and a shoulder near 460 nm, similar to that reported by Moghaddasi et al.^16^ However, when the AgNO_3_ solution reacted with the curcumin solution, *C*-AgNPs were formed, and the UV–vis absorption spectra of *C*-AgNPs localized at 408 nm (Figure 3B) attributed to surface plasmon resonance.^10^ Furthermore, Figure 3A also shows that the UV–vis spectra of the AgNO_3_ solution have no absorption peaks in the range of 300–600 nm. According to the research results of Phanjom et al.,^17^ the UV–vis spectra of the AgNO_3_ solution have a shoulder near 220 nm in the ultraviolet region, which is consistent with the results of N. Jayaprakash et al.^18^Figure 3C displays the XRD pattern of *C*-AgNPs prepared using a nanocurcumin solution. The 2θ values of 38.25, 44.50, and 65.035° corresponding to the (111), (200), and (220) planes, respectively, indicate that the as-prepared AgNPs were similar to those obtained in a previous work.^10^ The crystal structure parameters of *C*-AgNPs for the space group *Fm*3̅*m* calculated from the Rietveld refinement of XRD data were *a* = *b* = *c* = 4.075 Å. As shown in Figure 3D, the FTIR band absorption characteristic of the *C*-AgNPs shows localized peaks at 517, 1101, 1639, 1736, 2876, 2924, and 3445 cm^–1^. The 517 cm^–1^ peak is related to *C*-AgNPs banding with oxygen from hydroxyl groups of nanocurcumin.^9,19^ The 3445 cm^–1^ peak is assigned to the O–H stretching vibration, indicating the presence of hydroxyl groups in the reducing agent.^20^ Other peaks such as 1101, 1639, 1736, 2876, and 2924 cm^–1^ are attributed to the characteristic peaks of the nanocurcumin.^21^ TEM micrographs showed the particle size and morphology of *C*-AgNPs as can be observed in Figure 3E. The distribution and particle size of *C*-AgNPs are shown in the inset of Figure 3E. It showed that the majority of *C*-AgNPs were in the size range of 5–25 nm, with an average size of 12.1 nm.

### 3.2 Antibacterial and Antifungal Activities of *C*-AgNPs

**Figure 4:**
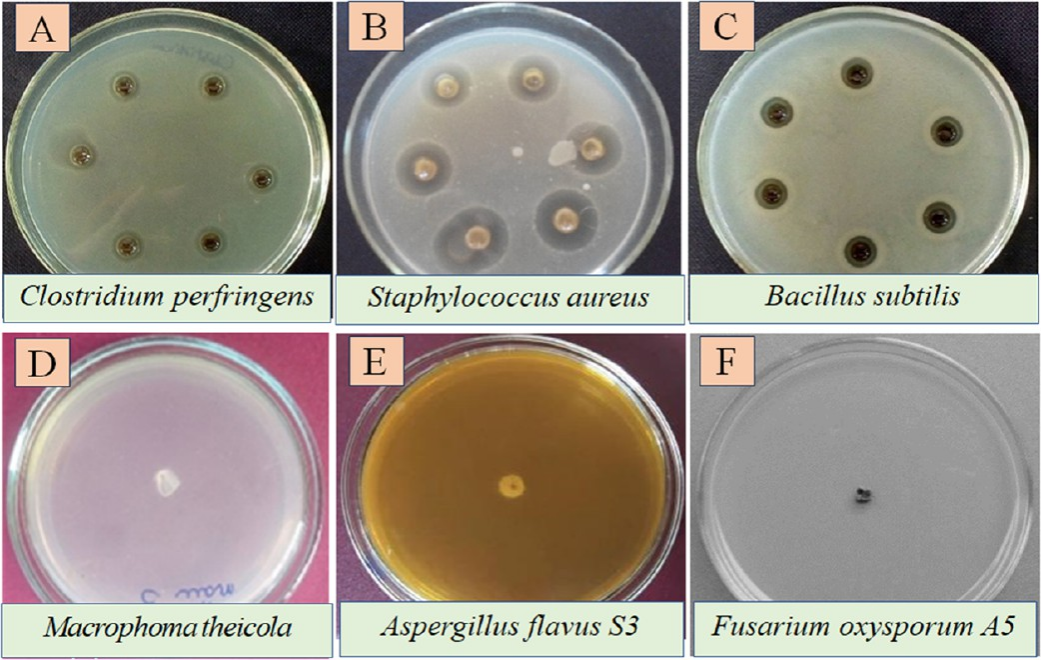
(A–C) Antibacterial activity of AgNPs against: (A) *C. perfringens*, (B) *S. aureus*, and (C) *B. subtilis*. (D–F) Antifungal activity of AgNPs against: (D) *M. theicola*, (E) *A. flavus* S3, and (F) *F. oxysporum* A5.

The antimicrobial activity was assayed at 30 ppm of *C*-AgNPs, and the inhibition zone formed and is shown in Figure 4. The antibacterial activities of *C*-AgNPs were tested via a diffusion method against three bacteria: *C. perfringens* (Figure 4A), *S. aureus* (Figure 4B), and *B. subtilis* (Figure 4C). The produced *C*-AgNPs displayed a very high zone of inhibition of 21.7 + 0.1 mm against *S. aureus*, which is higher than *C. perfringens* (zone of inhibition of 14.3 + 0.2 mm) and *B. subtilis* (zone of inhibition of 15.6 + 0.2 mm) (Figure 4A–C). The antibacterial properties of *C*-AgNPs are due to the release of Ag^+^ ions, which react with electron donor groups in oxygen- or nitrogen-containing molecules inducing cell death.^22^ The antifungal activity of *C*-AgNPs against *M. theicola*, which was isolated from mandarin peels collected from agricultural lands of rural villages of Thua Thien Hue province in Vietnam, is presented in Figure 4D. The potent antifungal effects on *M. theicola* B1 are similar to those found in the study by Khatoon et al.^23^

The potent antifungal effects against *A. flavus* S3 and *F. oxysporum* A5 are shown in Figure 4E,4F, respectively. Sondi and Salopek-Sondi^24^ indicated that the bactericidal activity of silver ions is primarily due to their interaction with the cytoplasm in the interior of the cells. It is well-known that the *C*-AgNPs penetrate through ion channels without causing damage to the cell membranes and then denature the ribosome and suppress the expression of enzymes and proteins essential for adenosine triphosphate production.^24,25^ This result may be attributed to the dissolution of the cellular contents in the culture broth, the disruption of the cell membrane structures with the loss of membrane permeability, and the inability to sustain adenosine triphosphate production; all of these processes are necessary for maintaining membrane dynamics.^25,26^ Based on the above results, we can conclude that silver nanoparticles have a commercial potential to be used in the prevention of food spoilage and in postharvest management to minimize decay until the consignment reaches its destination. We believe that, based on *C*-AgNPs’ good antibacterial and antifungal properties, combining them with natural polymer films can optimize the antibacterial activity of the biodegradable nanocomposite film.

### 3.3 Properties and Applicability of Nanocomposite Films

**Figure 5:**
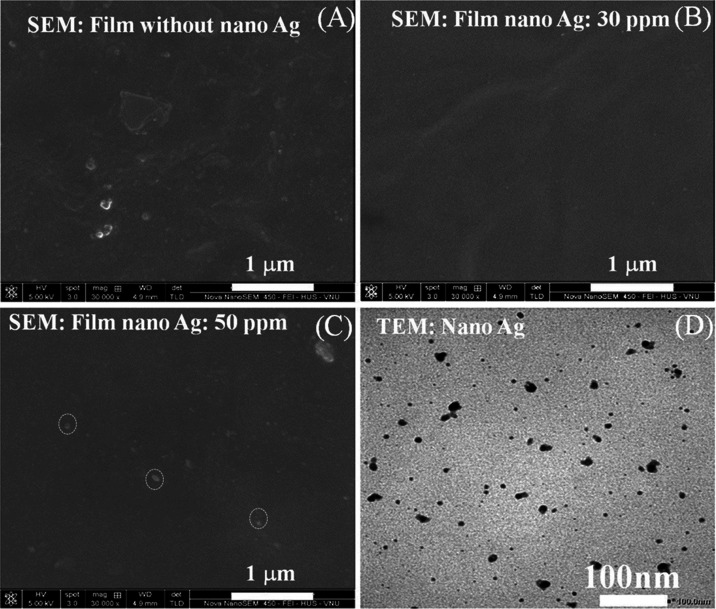
Surface morphologies of nanocomposite films: (A) without *C*-AgNPs; (B) *C*-AgNPs: 30 ppm; (C) *C*-AgNPs: 50 ppm; and (D) surface morphologies of *C*-AgNPs.

The microstructure of the fabricated films was investigated by SEM imaging, as shown in Figure 5. It can be seen in Figure 5 that the surfaces of nanocomposite films were homogeneous, rendering the films flexible, and were easily removed from the flat glass plate after drying at a temperature of around 25 °C (Figure 5C–D). SEM images showed that the surface of active nanocomposite films had remarkable differences. In Figure 5A, the absence of the AgNPs causes a discontinuous structure with lipid droplets embedded in the polymer network. However, a smooth uniform regular surface was observed in all samples containing *C*-AgNPs (Figure 5B–C). There was no phase separation between PVA, Ag nano, chitosan, and starch. The nanocomposite film containing AgNPs at 30 ppm concentration showed well-shaped microstructures. In addition, a close look at Figure 5C shows that silver nanoparticles (light streaks) lying on the surface of the nanocomposite film are very similar to the distribution of silver nanoparticles in the *C*-AgNP solution (TEM image in Figure 5D).

**Figure 6:**
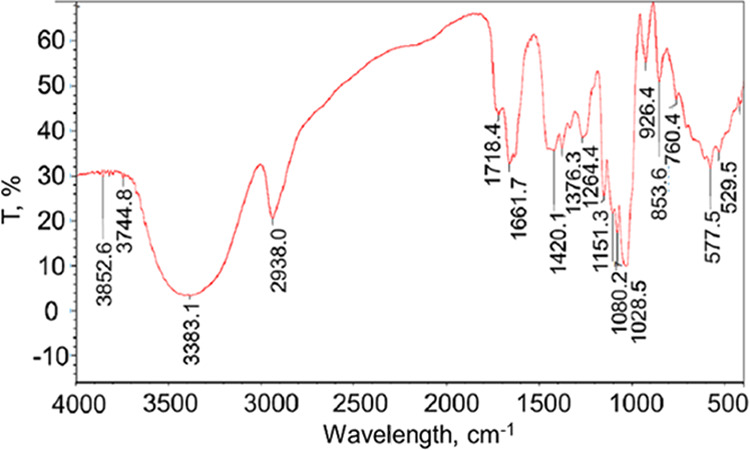
FTIR spectra of compounds present in the nanocomposite films formed with 30 ppm of AgNPs.

The functional groups present in the compounds of the nanocomposite films made with 30 ppm of *C*-AgNPs were studied using Fourier transform infrared spectroscopy (FTIR) in the range of 4000–500 cm^–1^ (Figure 6). The nanocomposite film showed absorption peaks at 1661.7 and 1376.3 cm^–1^ related to amide I and II of C=O stretching,^27^ N–H/C–N stretching, and CH_2_ wagging coupled with OH groups of chitosan, respectively.^28^ The peak observed at 1420.1 cm^–1^ is due to CH_2_ bending, and the peak at 2938 cm^–1^ is a characteristic of the −CH_2_ asymmetric stretching of PVA.^28^ The absorption peak observed at 3383 cm^–1^ indicates the hydrogen-bonding nature of the OH/NH_2_ stretching. The silver nanoparticles loaded nanocomposite film has shown the above characteristic peaks with a slight shift of the peak from 1264.4 to 1420.1 cm^–1^ corresponding to the amide III band.^28^ The Ag ions and electron-rich groups of NH_2_ and OH groups formed co-ordination bonds; the stretching vibration at 3383.1 corresponding to OH/NH_2_ groups shifted to 3852.6 cm^–1^, indicating that the silver particles are bound to the functional groups present both in chitosan and in PVA.^27^ In a study, the stretching vibrations of the −OH bond of the prepared chitosan were found at 3478.68 cm^–1^ and that for C–H was observed at 2924.13 cm^–1^.^29^ All of the above observations found in the IR spectra of films confirmed the presence of silver nanoparticles in the nanocomposite film networks.

**Figure 7:**
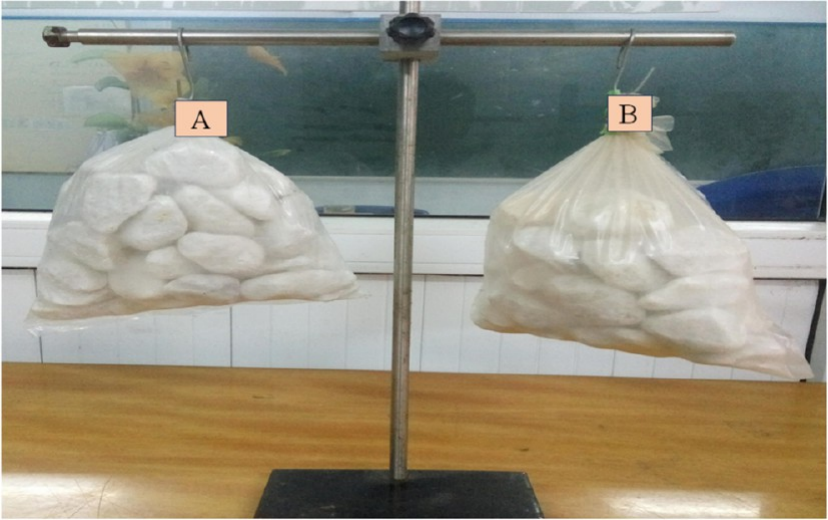
Comparison of the biodegradable bags prepared from the nanocomposite films with conventional PE plastic bags: (A) conventional PE plastic bags; and (B) biodegradable bags using nanocomposite films.

In the process of material synthesis, the mechanical properties are important for nanocomposite films. It is essential to evaluate the material properties for food packaging applications. The mechanical properties, such as tensile strength (TS), Young’s modulus (YM), and elongation at break (EB), can have determining effects on the quality of food packaging materials.^30^Figure 7 shows a comparison of the biodegradable bags (Figure 7B) prepared from the nanocomposite films along with conventional PE plastic bags (Figure 7A). They contained 2 kg of stone and were kept for 12 months. Therefore, we qualitatively compared the load-carrying capacities of these two bags. With this intention, we can observe the reactivity of the biodegradable bags that were fabricated.

**Figure 8:**
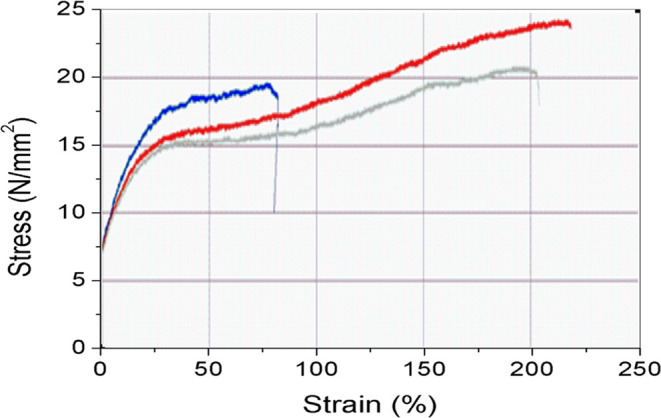
Stress–strain curve of the nanocomposite film containing AgNPs at 30 ppm.

Figure 8 shows the stress–strain curve of the nanocomposite film. The addition of a proper amount of silver nanoparticles to the nanocomposite film increases the tensile strength of resulting films. This could be explained by the homogeneous dispersion of silver nanoparticle layers in a matrix consisting of chitosan, cassava starch, and PVA. In other words, such an improvement in the mechanical strength in the nanocomposite materials is related to the adequate dispersion of Ag nanoparticles that act as reinforcing fillers like that reported by.^31^ The maximum values for stress strength and strain of the film were 24 N/mm^2^ and 220%, respectively. This is consistent with published references.^31,32^ The tensile strength of the packaging film should be greater than 3.5 N/mm^2^, according to conventional standards.^33^ This means that the nanocomposite film prepared in this research has enough mechanical strength to be used as a food packaging film, which is equivalent to the mechanical strength of conventional membranes.

**Figure 9:**
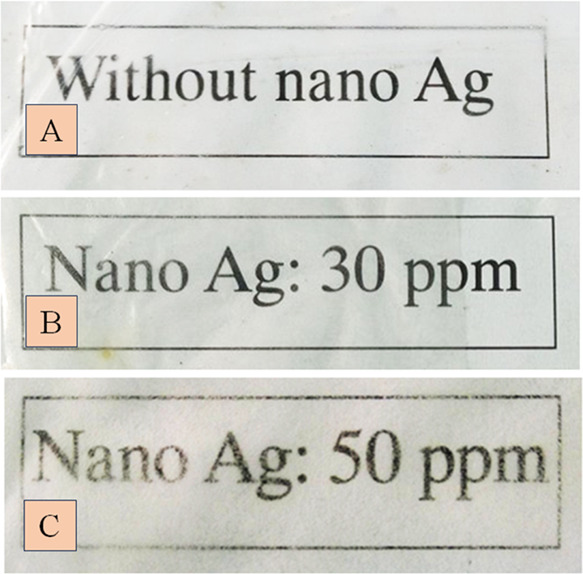
Digital images of nanocomposite blend films: (A) nanocomposite film without *C*-AgNPs; (B) nanocomposite film containing 30 ppm of *C*-AgNPs; and (C) nanocomposite film containing 50 ppm of *C*-AgNPs.

The nanocomposite films (Figure 9A,B) were found to be highly flexible, smooth, and transparent. At a high concentration of *C*-AgNPs (50 ppm), the nanocomposite films (Figure 9C) became less transparent, heavier, and thick with a rough surface. This observation was consistent with the study of the microstructure of the nanocomposite films, as shown in Figure 5B.

**Figure 10:**
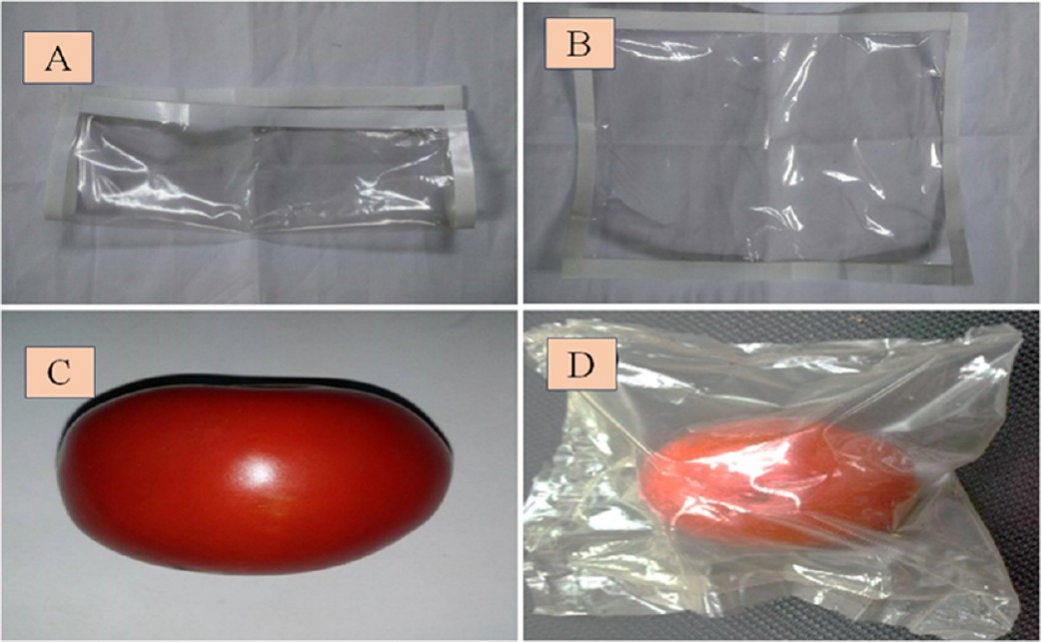
Bag made of nanocomposite films containing AgNPs at 30 ppm: (A–B) the bag shape is made from a biodegradable film; (C) image of tomatoes harvested in Thua Thien Hue province; and (D) image of tomatoes preserved by biodegradable bags.

As presented in Figure 10, the fabricated biodegradable bag contains natural and degradable resources via a green production approach, thereby reducing our dependence on fossil fuels. This bag can be used to retain the taste and texture of food to maintain the quality and safety of products during transport and storage, as well as to extend its viability by preventing unwanted effects caused by microorganisms, chemical contaminants, oxygen, moisture, and light.^34^

In recent times, there has been a surging interest in the advancement of biopolymers, which are polymers derived from natural sources, owing to their low toxicity and environmentally friendly properties.^6,7^ Nanoparticles offer a versatile solution for creating packaging and coating materials in the food industry.^5,11^ In this particular study, the fruits possess an outer protective layer, making nanosilver not only nontoxic to the fruits but also capable of safeguarding them against pathogens caused by bacteria and fungi, as explained above. For agricultural products lacking a protective covering, employing bags made of nanocomposite films (as shown in Figure 10) would be a suitable alternative.

**Figure 11:**
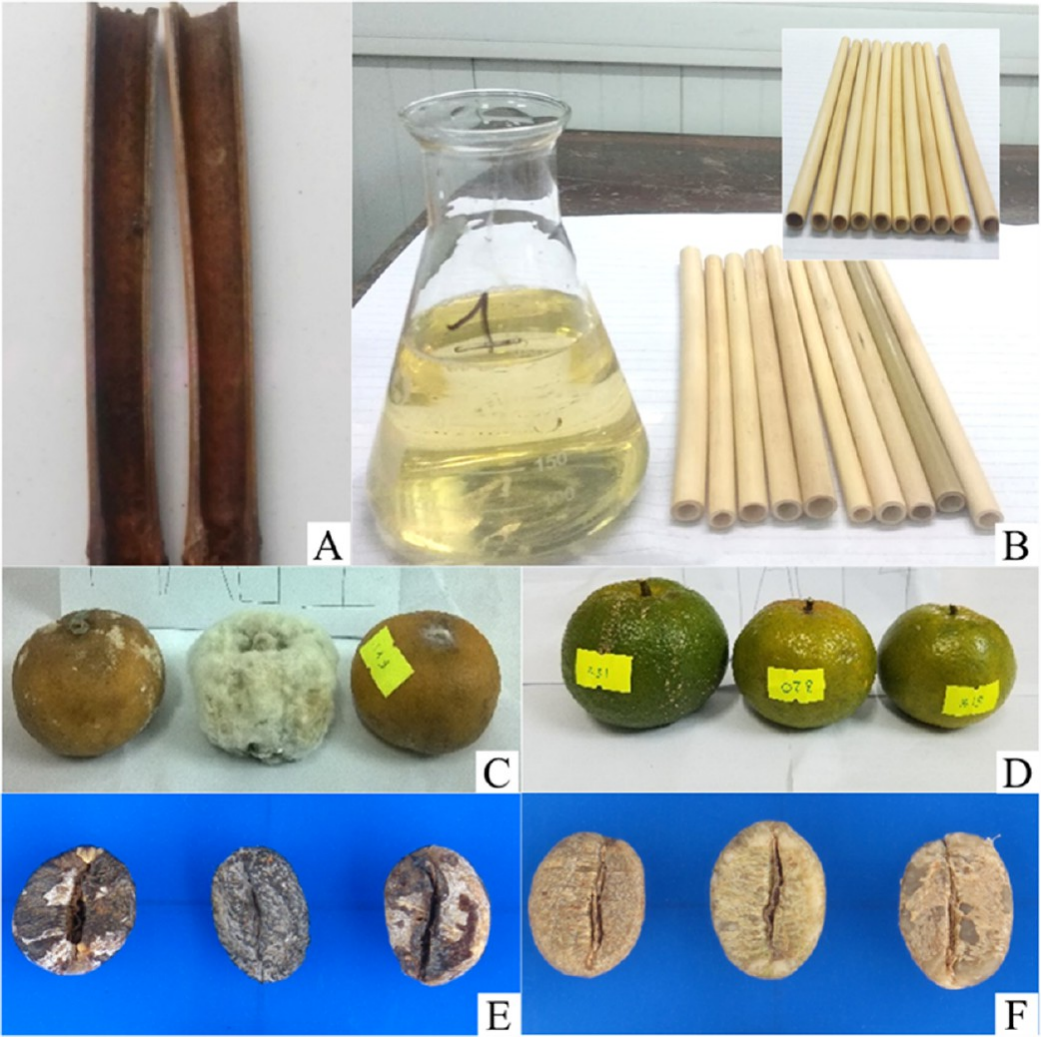
(A) Bamboo straws infected with *A. flavus* fungus; (B) bamboo straws protected with nanosilver-chitosan solution after 15 days; (C) mandarin (Citrus deliciosa Tenore) infected with *M. theicola* fungus; (D) mandarin protected with nanosilver-chitosan solution after 30 days; (E) coffee beans infected with *A. flavus* fungus; and (F) coffee beans protected with nanosilver-chitosan solution in the same condition.

Our recent research has shown that the Ag-chitosan solution is very effective in protecting coffee beans and bamboo straws from *A. flavus* fungus as shown in Figure 11B compared to no-treated straw, where *A. flavus* fungus grows strongly after 15 days at room temperature (Figure 11A). Figure 11C shows mandarin infected with *M. theicola* fungus, whereas Figure 11D shows mandarin protected with a nanosilver-chitosan solution in the same condition. It shows that the 50 ppm AgPNs can be applied as a preservative for mandarin, leading to the storage time of the sample increasing by up to 35 days at room temperature (Figure 11D).^35^ If a preservative solution is not used, the mandarin can only be used for 5–7 days, and after 2 weeks, it will be infected with its entire outer skin (Figure 11C). Furthermore, the mandarin protected with a nanosilver-chitosan solution lasted 35 days, not only retaining its beautiful shape and color but also its quality was guaranteed, such as the loss of mass was low (4.81%), the total sugar levels were about 10.5 mg/g compared with the levels of the control samples after 7 days of storage (11.12 mg/g), and the content of vitamin C was about 30 mg/100g. The same trend was observed for coffee beans infected with *A. flavus* fungus (Figure 11E) and coffee beans protected with nanosilver-chitosan solution in the same condition (Figure 11F). Thus, the *C*-AgNPs-chitosan solution can be used to protect agricultural products from pathogens caused by bacteria and fungi to improve their value and use quality.

**Figure 12:**
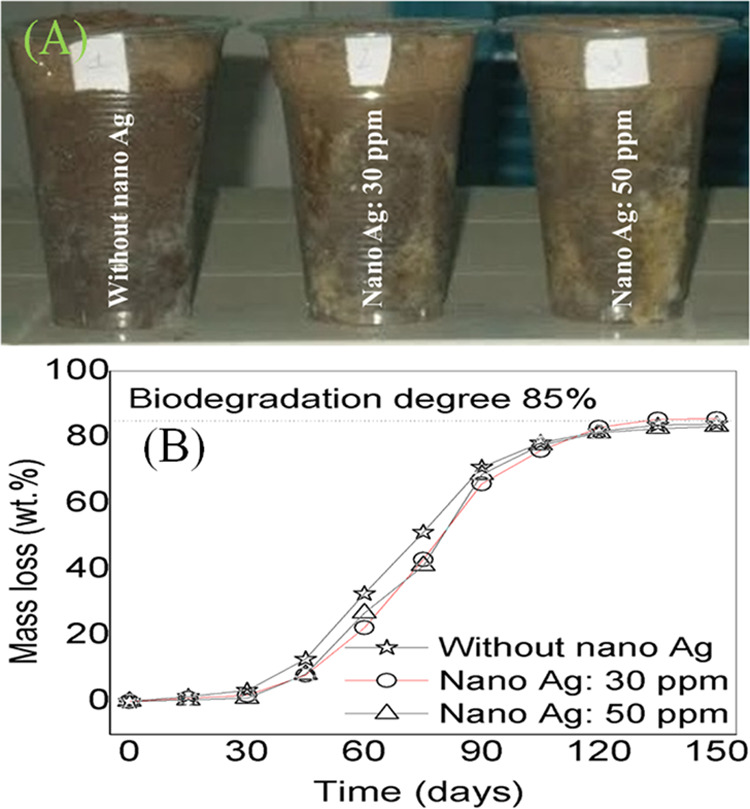
(A) Photographs of the observed samples, and (B) mass loss (as an indicator of the biodegradation) of nanocomposite films.

The mass loss (as an indicator of the biodegradation) observation for the prepared films is presented in Figure 12A. The nanocomposite films were decomposed approximately 85 wt % within 120 days after being buried in the soil (Figure 12B). The use of nanocomposite films shows the rational use of natural resources and reduces environmental pollution. The reason is the biocompatibility of the nanocomposite films and soil microorganisms, which promotes the decomposition of the films.

## 4 Conclusions

In this study, *C*-AgNPs were synthesized using a nanocurcumin solution, which indicated the role of nanocurcumin as a reducing and stabilizer agent. The synthesized *C*-AgNPs were found to have particle sizes in the range of 5–25 nm. They showed good antibacterial activity against *C. perfringens*, *S. aureus*, and *B. subtilis* and antifungal activity against *M. theicola*, *A. flavus*, and *F. oxysporum*; thus, they can be used to protect coffee beans, bamboo straws, and mandarin from these pathogens to improve their value and use quality. The incorporation of silver nanoparticles, chitosan, cassava starch, and PVA was found to be useful and simple for producing nanocomposite films by a casting method. The nanocomposite films were found to be highly flexible, smooth, and transparent. The maximum values for stress strength and strain of the film are 24 N/mm^2^ and 220%, respectively. This means that the nanocomposite film prepared in this work has enough mechanical strength to be used for commercial packaging. Besides, the mass loss of nanocomposite films (i.e., biodegradation) after 120 days of being buried in the soil was 85 wt %. This indicates that the films made by natural biodegradable materials can reduce environmental pollution and landfill.
